# Loneliness as a mediator of social relationships and health-related quality of life among refugees living in North Rhine-Westphalia, Germany

**DOI:** 10.1186/s12889-021-12303-5

**Published:** 2021-12-08

**Authors:** Matthias Hans Belau, Heiko Becher, Alexander Kraemer

**Affiliations:** 1 School of Public Health, Bielefeld University, Bielefeld, Germany; 2 Institute of Medical Biometry and Epidemiology, University Medical Center Hamburg-Eppendorf, Hamburg, Germany; 3 University Medical Center Hamburg-Eppendorf, Institute of Medical Biometry and Epidemiology, Martinistraße 52, D-20246 Hamburg, Germany

**Keywords:** Health-related quality of life, Refugees, Social relationships, Loneliness, Mediation

## Abstract

**Background:**

Since 2015, more than one million people fled to Germany – mainly from war-affected countries. Nevertheless, little is known about social determinants in refugees located in Germany. This study aims to test the mediation effect of loneliness between social relationships, comprising social integration and social support, and health-related quality of life among refugees living in North Rhine-Westphalia, Germany.

**Methods:**

The investigation utilizes data from the FlueGe Health Study (N=326), a cross-sectional study conducted by Bielefeld University. The data was collected between February and November 2018 and included interviews and examinations. Participants were recruited from shared and private accommodation in several cities in North Rhine-Westphalia, Germany. We first analyzed correlations between social integration, social support, loneliness, and physical and mental component of health-related quality of life. We then conducted mediation analyses using structural equation modeling.

**Results:**

The majority of respondents were socially isolated, perceiving a moderate degree of loneliness and social support. In addition, the physical and mental components of health-related quality of life indicate that participants predominantly experienced mental rather than physical impairments. Results from mediation analyses showed indirect effects of loneliness on the association between social integration and mental health (ß = 0.495, 95% bias-corrected and accelerated confidence interval (BCa CI) = [0.018, 0.972]), and between social support and both physical (ß = 0.022, 95% BCa CI = [0.004, 0.040]) and mental health (ß = 0.067, 95% BCa CI = [0.037, 0.097]).

**Conclusions:**

Loneliness played a mediating role in the association between social relationships and health-related quality of life among refugees living in North Rhine-Westphalia, Germany. The results provide implications for both, health policy and the host society.

**Supplementary Information:**

The online version contains supplementary material available at 10.1186/s12889-021-12303-5.

## Background

In 2015 and 2016, Germany was the main country of asylum for people fleeing from war-affected countries such as Syria, Iraq, Afghanistan, and Central Africa, with 441,900 and 722,400 asylum applications, respectively [1]. Many of them have been subjected to stressful and adverse experiences before, during, and after the flight [2]. Experiences of war-related violence and torture in the countries of origin, traumatic events during the journey such as physical assault, extortion, and sexual violence as well as the stressors of the asylum process and poor living conditions in the host country lead to a significant burden of mental illness such as post-traumatic stress disorder (PTSD), depression, and anxiety [3–5]. Concurrently, there are often barriers to receiving medical services and accessing the social system [6, 7]. In Germany, refugees also have limited access to medical care during their asylum process [8]. Therefore, generalized resistance resources [9], including social relationships [10], are needed to deal with those health-related challenges. Literature reveals that social relations have an impact on refugees’ health and well-being [11, 12]. However, many refugees are dealing with loneliness and the experience of loss of friends and family members throughout the migratory journey to Germany [2]. In some cases, family members are left behind to seek asylum in the hope of eventual reunification [2]. The family in particular, as an element of resilience, is assigned a central role in promoting and maintaining health and well-being [13].

Little is known concerning the social relationships of refugees living in Germany and its impact on subjective health parameters such as health-related quality of life (HRQoL). HRQoL captures the personal and social dimensions of a person’s well-being and is considered to be a valid, reliable, and robust measure of health status [14] in migrant populations [15]. In addition, HRQoL has been shown to be a significant predictor of health care utilization [16] and mortality [17], underscoring its importance for health policy and public health with respect to refugees. A study focusing on refugee women from war-affected countries resettled in Germany revealed that HRQoL was moderate and significantly worse than that of the European population [18]. Another study assessing HRQoL among refugees in Germany using survey data showed that ethnic groups of refugees (Syrians, Afghans, and Eritreans) differ inherently in their HRQoL [19]. Studies examining the social determinants of refugees’ HRQoL during settlement in Germany are lacking. To the best of our knowledge, no study so far aimed to explore the (perceived) health effects of social relationships among refugees resettled in Germany. Literature shows that refugees’ family structures vary considerably [20]. The loss of family members and friends seriously affects their loneliness scores. Loneliness, in turn, is a predictor of mental health problems among refugees, such as depression [21].

A theoretical model by Wilson and Cleary [22] considers and describes the influence of the individual and the environment on various dimensions of HRQoL. Social environmental characteristics are the interpersonal or social influences on health and well-being, including the influence of family and friends [23]. Several studies have shown that HRQoL is closely linked with social relationships comprising social networks [24] and social support [25] as an interconnected term. There is evidence that social isolation and lack of social support can lead to loneliness [26]. Few studies postulate an association between lack of social integration and loneliness [27], while loneliness is linked to HRQoL [28]. Thus, the relation between HRQoL and social relationships could be mediated by loneliness. A study assessing the interrelationships among perceived social support, loneliness, and HRQoL [29]showed that loneliness mediates the relationship between social support and HRQoL among South Korean older adults [29], but none in a refugee population.

The goal of our study was to test the mediation effect of loneliness between (indicators of) social relationships, measured on two separate scales as social integration and as social support, and HRQoL among refugees living in North Rhine-Westphalia, Germany. We hypothesized that refugees will develop loneliness in the migration process due to the loss of friends and family members in their country of origin. To test the mediation hypothesis, a recursive model was adopted, and results are based on data from the “FlueGe Health Study” (FHS).

## Methods

### Participants and data collection

The present investigation utilizes data from the FHS, a cross-sectional study administered by the research class “FlueGe – refugee health” at the School of Public Health at Bielefeld University [30]. The FHS aimed to provide health data of refugees from the main countries of origin that contributed to the European refugee crisis in 2015 and 2016 in the region of East Westphalia-Lippe in North Rhine-Westphalia, Germany. The data was collected between February and November 2018 and included personal interviews and physical examinations, carried out by trained interviewers. The interview questionnaire was translated into five languages: Arabic, Farsi, Kurmanji, English, and German. The translation followed the scientific standard [31, 32]. First, certified translators by Kantar Public, a consulting and market research institute, translated the questionnaire from the original version into the remaining languages. Subsequently, other native Arabic, Persian, and Kurdish speakers who were simultaneously fluent in English and German blindly back-translated the translated questionnaire into the original language. The back-translation was done both literally and semantically. Participants were recruited from shared and private accommodation. Municipal cooperation partners and social workers provided access to potential participants. The FHS included all participants who were willing to participate, except they were younger than 18 years of age, could not speak Arabic, Kurmanji, Farsi, English, or German, or if they have been in Germany for more than five years. A total of 827 men and women aged 18 to 75 years were assessed for eligibility and invited to the study. Of these, 130 individuals had an inadequate language level, and 371 individuals refused to participate in the FHS. The main reasons were personal reasons as well as having no interest in the research. Overall, 326 men and women signed informed consent and completed the study. Approval from the Ethics Commission of Bielefeld University was obtained before the data was collected to ensure ethical and data protection guidelines.

### Measures

Short Form-12 Health Survey-SOEP (SF-12-SOEP) [33] was used, assessing HRQoL. The information was aggregated into a physical component summary (PCS) and a mental component summary (MCS) score. To compare to published means, both scales were transformed into a range from 0 (minimum) to 100 (maximum), and higher values indicate a better state of health. Furthermore, norm-based scoring was performed by first z-transforming SF-12-SOEP scales using factor loadings on PCS and MCS for weighting served by the SOEP2004 data as the norm population [33] and then transforming them to a mean value of 50 and a standard deviation (SD) of 10. Cronbach`s alpha of PCS and MCS in the current study were 0.85 and 0.83, respectively.

Social integration was measured according to the Social Network Index (SNI) [34, 35]. The SNI contains three domains, each scored from 0 to 2: (i) cohabitation with spouse or partner, (ii) contacts with close friends and family, and (iii) affiliation with the religious community and voluntary associations. Cohabitation was scored as 2 if the participant reported living with spouse or partner, and 0 if not. The frequency of contact with close friends and family (face-to-face or by phone, at least once a month) was scored 2 (≥12 contacts), 1 (3 to 11 contacts), or 0 (<3 contacts). Affiliation was scored 2 if a participant was a member of a religious group (attending services and activities at least once a month) and member of a group without religious affiliation (sports, community, political, or professional associations common in Germany) or at least member of two groups without religious affiliation. Participants were given a score of 1 if they were a member of a religious group or member of a group without religious affiliation, and a score of 0, if participants weren’t a member of a religious group or a group without religious affiliation. The SNI ranges from 0 to 6, a score of 0 to 1 indicates strong social isolation (low degree), a score of 2 to 3, 4 to 5, and 6 indicates a moderate, high, and a very high degree of social integration, respectively.

Social support was measured with the Medical Outcomes Study Social Support Survey (MOS-SSS) [36], which consists of four separate subscales (emotional/informational support, tangible support, affectionate support, positive social interaction) and an overall summary score. To compare to published means, all scales were transformed into a range from 0 (minimum) to 100 (maximum), and higher values indicate a better perception of social support. Cronbach`s alpha of emotional/informational support, tangible support, affectionate support, positive social interaction, and overall summary score for the present sample was 0.89, 0.92, 0.86, 0.90, and 0.94, respectively.

The three-item Loneliness Scale-SOEP (LS-SOEP) [37] was used to measure the degree of loneliness. Participants were asked about their agreement on the following questions: (i) “How often do you feel that you lack companionship?”, (ii) “How often do you feel left out?”, and “How often do you feel isolated from others?” Each question was answered based on a five-level rating scale: Never (0), seldom (1), sometimes (2), often (3), and very often (4). The information was aggregated into a summary score ranging from 0 (minimum) to 12 (maximum) with a higher score indicating stronger feelings of loneliness. Cronbach`s alpha of LS-SOEP in this study was 0.68.

Sociodemographic information included age, sex, country of origin, and education, which was inquired according to the Comparative Analysis of Social Mobility in Industrial Nations (CASMIN) classification [38]. First, nine educational groups were distinguished, which result from a combination of school and vocational qualifications. Then, the CASMIN index was used to categorize three groups: low (general elementary education and/or basic vocational qualification), medium (intermediate general qualification and/or intermediate vocational qualification), and high education (lower or higher tertiary education).

### Statistical analyses

**Fig. 1:**
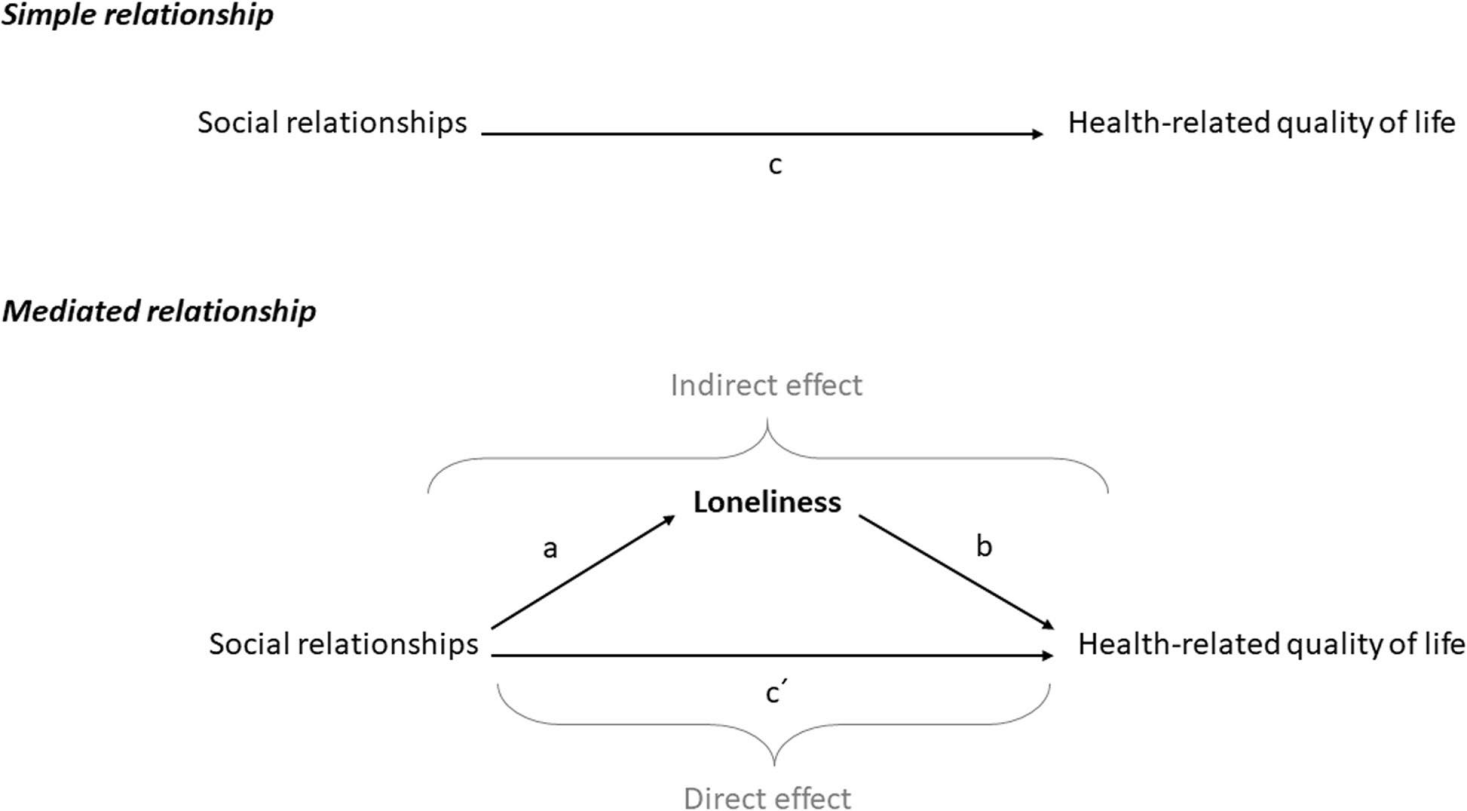
Simple mediation model of loneliness between social relationships and health-related quality of life

Statistical analyses were performed using STATA MP, version 16. Descriptive statistics were used to identify sample characteristics. To test our hypothesis, Spearman correlation and mediation analyses using structural equation modeling (SEM) following causal steps approach by Barron and Kenny [39] and comments by Preacher and Hayes [40, 41] were performed. A statistical diagram of the simple mediation model can be found in Fig. 1. In a linear regression model, the health-related quality of life measured as PCS (models 1.1 and 2.1) and MCS score (models 1.2 and 2.2) as metric outcome variables were regressed on loneliness (mediator variable) and both social integration (model 1.1 and 1.2) and social support (model 2.1 and 2.2) as predictor variables, respectively. Adjustments were made for age, sex, and education, which were regarded as potential confounders [42]. The adequacy of the structural models was evaluated according to the following model fit criteria: Chi-square statistic/degrees of freedom (χ^2^/df) < 3.0, root mean square error of approximation (RMSEA) ≤ 0.06, comparative fit index (CFI) ≥ 0.90, and Tucker-Lewis index (TLI) ≥ 0.90 [43]. Subjects presenting a missing value for at least one of the modeling variables were excluded from analyses (listwise deletion). We computed 95% bias-corrected and accelerated (BCa) confidence intervals (CI) from 10,000 bootstrap samples, as it yields accurate CI for indirect effects [44]. Mediation effects (indirect effect of predictor *X* on outcome *Y* through mediator *M*, defined as the product of *a* (*X* on *M*) and *b* (*M* on *Y*, controlling for *X*)) were considered significant if the 95% BCa CI does not contain zero.

## Results

Characteristics of the study population (N=326) are summarized in Table 1. Men constituted the majority of participants (73.0%), and the overall median age was 30.0 years. Syrians were the most represented group (40.6%), followed by Iraqis (24.3%), Afghans (12.9%), Africans (7.1%), and Iranians (5.9%). Respondents of African origin came from Algeria (8.7%), Eritrea (13.0%), Nigeria (26.1%), Somalia (17.4%), Ghana (13.0%), Guinea (4.4%), Morocco (8.7%), and Egypt (8.7%). More than three-fourths of the respondents have been in Germany for more than two years (76.0%), 60.7% reported a secure (entitlement to asylum, refugee protection, subsidiary protection, and a national ban on deportation), and 39.3% an insecure residence status (in procedure, temporary suspension of deportation, and a requirement to leave).

**Table 1:** Characteristics of participants by sex (N=326)

		All participants		Male		Female	
		**n**	**%**	**n**	**%**	**n**	**%**
**Age**, mean (SD)		32.4 (11.0)		31.9 (11.2)		33.8 (10.4)	
**Sex**		326	100.0	238	73.0	88	27.0
**Country of origin**	Syria	132	40.5	96	40.3	36	40.9
	Iraq	79	24.2	56	23.5	23	26.1
	Afghanistan	42	12.9	33	13.9	9	10.2
	Iran	19	5.8	14	5.9	5	5.7
	African countries	23	7.1	14	5.9	9	10.2
	Other countries	30	9.2	24	10.1	6	6.8
	*Missing values*	1	0.3	1	0.4	0	0.0
**Education**^**a**^	High	63	19.3	48	20.2	15	17.1
	Medium	127	39.0	97	40.8	30	34.1
	Low	135	41.4	93	39.1	42	47.7
	*Missing values*	1	0.3	0	0.0	1	1.1
**Social integration**, mean (SD)	Overall summary score	2.6 (1.4)		2.5 (1.4)		2.6 (1.3)	
	*Missing values*	12	3.7	12	5.0	0	0.0
**Social support**, mean (SD)	Overall summary score	58.4 (27.0)		55.4 (27.7)		66.7 (22.9)	
	*Missing values*	21	6.4	15	6.3	6	6.8
	Emotional, informational support	55.0 (29.5)		52.3 (29.8)		62.1 (27.6)	
	*Missing values*	13	4.0	10	4.2	3	3.4
	Tangible support	60.4 (37.2)		58.2 (38.2)		66.5 (33.8)	
	*Missing values*	11	3.4	7	2.9	4	4.6
	Affectionate support	62.7 (35.0)		57.3 (35.6)		77.5 (28.5)	
	*Missing values*	12	3.7	8	3.4	4	4.6
	Positive social interaction	60.4 (34.3)		56.8 (35.0)		70.5 (30.2)	
	*Missing values*	10	3.1	6	2.5	4	4.6
**Loneliness**, mean (SD)	Overall summary score	4.5 (3.1)		4.6 (3.1)		4.2 (3.0)	
	*Missing values*	11	3.4	8	3.4	3	3.4
**Health-related quality of life**, mean (SD)	Physical component score	50.8 (10.5)		51.8 (10.7)		48.3 (9.5)	
	*Missing values*	17	5.2	14	5.9	3	3.4
	Mental component score	43.0 (14.5)		43.3 (14.9)		41.7 (13.3)	
	*Missing values*	17	5.2	14	5.9	3	3.4

^a^ CASMIN classification (26); SD standard deviation; n quantity; % proportion

The majority of the participants reported a low (23.0%, men 22.7%, women 23.9%) and moderate degree (46.3%, men 46.2%, women 46.6%) of the SNI, indicating social isolation. Only 2.1% of male participants reported a very high degree of social integration. In addition, participants perceived a moderate degree of loneliness and social support. PCS and MCS scores indicate that participants predominantly experienced mental rather than physical impairments.

Table 2 provided Spearman’s correlation coefficients among the variables of interest. The results indicated that social integration was positively related to MCS score (r = 0.276, *p* < 0.001). Social support was positively (r = 0.312, *p* < 0.001) and loneliness negatively (r = -0.442, *p* < 0.001) correlated to MCS score. Correlations also emerged between MCS score and social support subscales (emotional/informational support (r = 0.197, *p* < 0.001), tangible support (r = 0.268, *p* < 0.001), affectionate support (r = 0.282, *p* < 0.001) and positive social interaction (r = 0.321, *p* < 0.001)). In addition, loneliness was negatively associated with social integration (r = -0.162, *p* = 0.007) and social support overall summary score (r = -0.394, *p* < 0.001) and subscales (emotional/informational support (r = -0.278, *p* < 0.001), tangible support (r = -0.343, *p* < 0.001), affectionate support (r = -0.337, *p* < 0.001) and positive social interaction (r = -0.400, *p* < 0.001)).

**Table 2:** Spearman correlation among social integration, social support, loneliness and HRQoL

	1	2	3	4	5
**Social integration**					
**1** Overall	1.000				
**Social support**					
**2** Overall	0.278***	1.000			
**Loneliness**					
**3** Overall	-0.162**	-0.394***	1.000		
**Health-related quality of life**					
**4** Physical component score	-0.117	0.085	-0.103	1.000	
**5** Mental component score	0.276***	0.312***	-0.442***	-0.032	1.000

* *p*<0.05, ** *p*<0.01, *** *p*<0.001

**Fig. 2:**
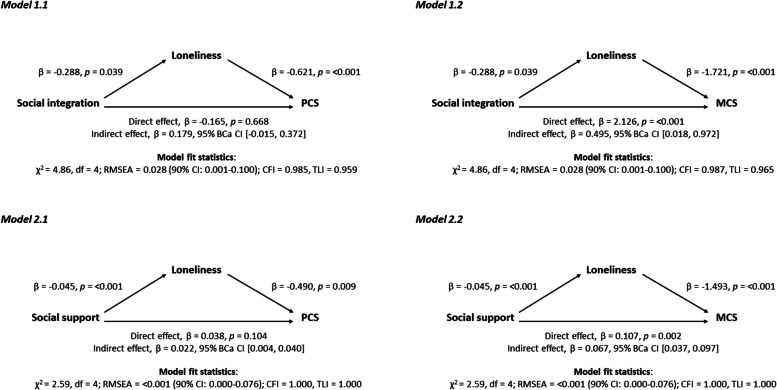
Models of indicators of social relationships as predictor variables of health-related quality of life, mediated by loneliness, adjusted for age, sex, and education. β: regression coefficient; BCa CI: bias-corrected and accelerated bootstrapped confidence interval based on 10,000 samples; CI: confidence interval; *p*: *p*-value; χ^2^: Chi-square statistic; df: degrees of freedom; RMSEA: root mean squared error of approximation; CFI: comparative fit index; TLI: Tucker-Lewis index

To assess mediation, we computed estimates of direct (c’), indirect (ab) and total effects (c = c’ + ab) between indicators of social relationships and HRQoL considering loneliness as mediator variable (see Fig. 2). In line with our hypothesis, mediation effects of loneliness between social integration and MCS (ß = 0.495, 95% BCa CI = [0.018, 0.972]), and social support (overall summary score) and both PCS (ß = 0.022, 95% BCa CI = [0.004, 0.040]) and MCS score (ß = 0.067, 95% BCa CI = [0.037, 0.097]) were found. Each mediation model showed an acceptable fit (see Fig. 2). Results including the total effects are shown in more detail in the Additional file 3. We also found indirect effects of loneliness between social support subscales and HRQoL. The indirect effect of loneliness on the association between emotional/informational support, tangible support, affectionate support, positive social interaction and PCS score was ß = 0.018 (95% BCa CI = [0.004, 0.031]), ß = 0.014 (95% BCa CI = [0.003, 0.026]), ß = 0.016 (95% BCa CI = [0.004, 0.028]) and ß = 0.016 (95% BCa CI = [0.002, 0.030]), respectively. For MCS score, mediation effects of loneliness were found for emotional/informational support (ß = 0.053, 95% BCa CI = [0.028, 0.078]), tangible support (ß = 0.044, 95% BCa CI = [0.022, 0.067]), affectionate support (ß = 0.046, 95% BCa CI = [0.022, 0.070]) and positive social interaction (ß = 0.052, 95% BCa CI = [0.026, 0.079]).

## Discussion

This study investigated the concurrent effects of structural and functional indicators of social relationships and loneliness on HRQoL and examined the mediating role of loneliness between indicators of social relationships and HRQoL among refugees living in North Rhine-Westphalia, Germany. The majority of respondents were affected by social isolation, perceiving a moderate degree of loneliness and social support. Our findings indicated that social integration, social support, and loneliness were all correlated with HRQoL. Regression analyses revealed that a lack of both social integration and social support were associated with poorer mental health status, which is consistent with findings of previous studies [12, 45]. In addition, a lack of social integration and social support were associated with higher levels of loneliness, while loneliness was related to worse physical and mental health. These findings are also consistent with other studies [46, 47]. Concerning mediation analyses, our results suggest that the perception of social support, and to some extent social integration itself, determine the level of loneliness and, through this pathway, influence refugees’ HRQoL.

Our results corroborate other studies and national surveys that show almost similar patterns of social integration, perceived social support, and loneliness among refugees. A study among refugees in Germany using survey data showed that on average, refugees knew two people (mostly family members and persons from the same country of origin) with whom they could share personal thoughts and feelings [48]. A study assessing perceived social support among Arabic-speaking refugees in Jordan and Germany showed moderate to high social support among respondents [49]. This study also showed that there is a positive effect for mental health issues such as depressive and PTSD symptoms. In previous studies, social integration and having social support are shown to be positively associated with HRQoL [50]. Nevertheless, it should be noted that being alone is not necessarily perceived as painful [51], but a lack of social support often is [52]. A study focusing on physical and mental HRQoL among refugees in Germany showed consistent results, with refugees experiencing mental rather than physical impairments [53]. The study also showed that refugees’ physical health was better compared to the general German population, but their mental health was significantly worse, which is consistent with our findings. A study examining loneliness among refugees in Germany found similar levels of loneliness compared to our population studied [54]. Interestingly, the study also shows that refugees in 2020 were about as lonely as they felt in 2016 and 2017. From a public health perspective, these findings show the need for observing how long loneliness persists among refugees resettled in Germany. Further data collection and analysis are needed.

To the best of our knowledge, there is only one study by Kang et al. [29] considering the mediating role of loneliness between perceived social support and HRQoL among South Korean adults. No study so far had examined the mediation effect of loneliness between structural as well as functional indicators of social relationships (social network, social support) and HRQoL among refugees. In our study, an indirect effect of loneliness on the association between perceived social support and PCS score was found. However, a direct effect between perceived social support and PCS score was not detected. As a result, this suggests that perceived social support does not affect physical HRQoL independent of the effect of loneliness on physical HRQoL. Moreover, the indirect effect of perceived social support on both PCS and MCS scores through loneliness was found to be strong, suggesting that a lack of perceived social support promotes the experience of loneliness, which can have a detrimental impact on HRQoL. Thus, loneliness can play a critical role in the relationship between perceived social support and HRQoL among refugees. The results of this study suggest that professionals, policymakers, and the host society must give more attention to the causes of social isolation and loneliness in refugees and continue to invest in family reunification, language education as well as housing and labor market access. These aspects in particular are key factors for the social integration of refugees resettled in Germany and can help decrease loneliness and psychological distress [55].

### Limitations

Our study faces some limitations. We utilized data from the FHS, and selection bias might be an issue especially because participants in the FHS were self-selected. This means that individuals who were not interested in health issues have decided not to participate in the FHS. Moreover, language or health barriers may have hindered participation. Another limitation results from the different language versions of the items used. Particular attention was paid during translation to ensure the correctness of content and language, completeness, comprehensibility, and consistency. Nevertheless, measurement errors can occur [56]. The most widespread Kurdish language, Kurmanji, solely has a large number of different dialects and linguistic peculiarities [57]. Another limitation arise in connection with the cross-sectional design, which restricts the interpretation of the results of the mediation analysis [41]. We adopted a recursive model in which social isolation precedes loneliness and, through this pathway, influences HRQoL. Note that poor health can lead to social isolation and vice versa, e.g., due to confinement in bed or mental disorders such as depression. Therefore, a longitudinal study following the refugees’ social relationships, loneliness and HRQoL with time could be elucidating for furthers analysis. Furthermore, other explanations for associations in the mediator model are conceivable: Loneliness as a mediator may only be a correlate to the actual mediator, e.g., socioeconomic living conditions, which are not included in the model. It could also be possible that the mediator considered is influenced by the dependent variable (HRQoL), as other studies [58, 59] suggest. Since our data only included a convenience sample of 326 refugees from East-Westphalia-Lippe results cannot be generalized to all refugees in North Rhine-Westphalia and Germany. Finally, it is important to note that further studies need to incorporate more variables, such as socioeconomic status [60] and living conditions [61] that are positively associated with refugees’ HRQoL.

## Conclusions

This is the first investigation of testing the mediation effect of loneliness between indicators of social relationships and HRQoL among refugees by using SEM. Furthermore, our study provides information on social integration, loneliness, and perceived social support among refugees from war-affected countries after their resettlement in Germany. In conclusion, the majority of respondents were affected by social isolation, perceiving a moderate degree of loneliness and social support. In mediation analyses, loneliness played a mediating role in the association between social integration and MCS as well as perceived social support and PCS as well as MCS score. Together these findings suggest that loneliness can play a critical role in the relationship between perceived social support and HRQoL among refugees. The results of the study provide implications for both, health policy and the host society, respectively. Health policy has to foster refugees’ social and economic integration and the host society must be open and inclusive in its orientation towards cultural diversity to reduce loneliness and strengthen social relationships and HRQoL among refugees.

## Supplementary Information

**Additional file 1: FigureA. **Distribution plots of the variables of interest.

**Additional file 2: Figure B. **Two-way scatter plots among variables of interest.

**Additional file 3: TableA1. **Direct, indirect, and total effects, adjusted for age, sex, and education (*n *= 268).

## Data Availability

The data that support the findings of this study are available from Bielefeld University, but restrictions apply to the availability of these data, which were used under license for the current study, and so are not publicly available. Data are however available from the corresponding author (Matthias Belau/e-mail: m.belau@uke.de) upon reasonable request and with permission of Bielefeld University.

## Abbreviations

BCa: bias-corrected and accelerated
CASMIN: Comparative Analysis of Social Mobility in Industrial Nations
CI: confidence interval
FHS: FlueGe Health Study
HRQoL: health-related quality of life
LS-SOEP: Loneliness Scale-SOEP
MCS: mental component summary
MOS-SSS: Medical Outcomes Study Social Support Survey
PCS: physical component summary
SEM: structural equation modeling
SD: standard deviation
SF-12-SOEP: Short From-12 Health Survey-SOEP
SNI: Social Network Index
SOEP: Socio-Economic Panel
