# Supplementary figures and images for: Loneliness as a mediator of social relationships and health-related quality of life among refugees living in North Rhine-Westphalia, Germany
Source: BMC Public Health. 2021 Dec 8;21:2233. doi: 10.1186/s12889-021-12303-5 (PMC8656054; doi:10.1186/s12889-021-12303-5)

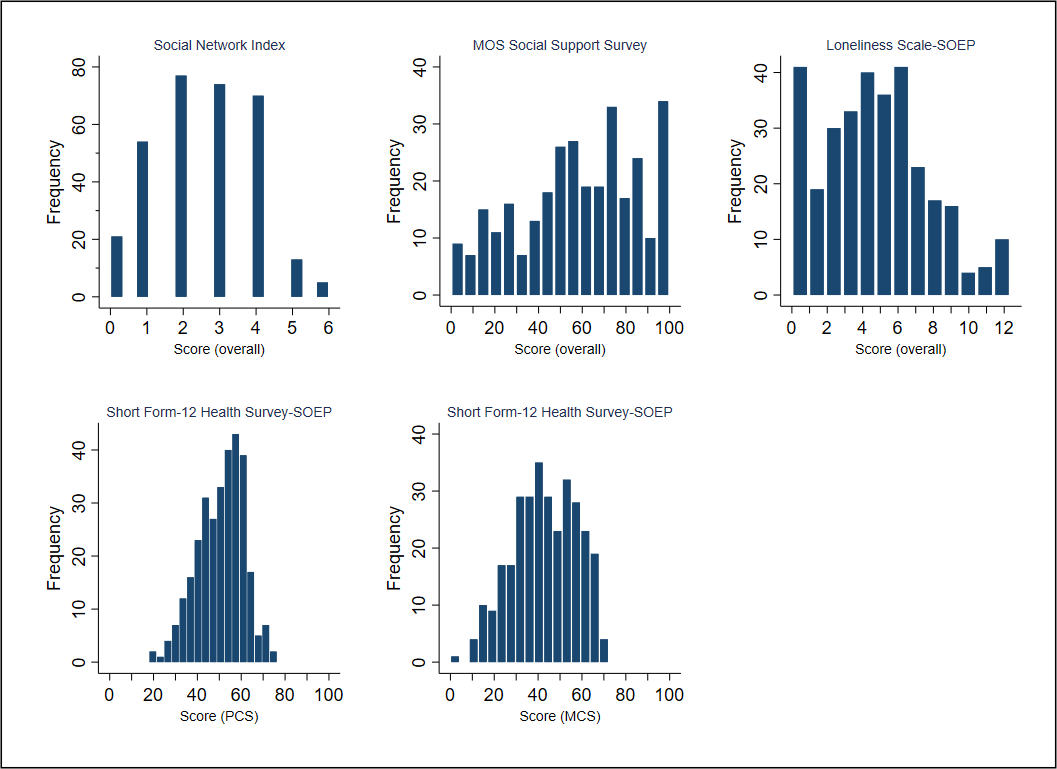

Supplement: Supplementary file 1 — Additional file 1: FigureA. Distribution plots of the variables of interest. [file 12889_2021_12303_MOESM1_ESM.tif]

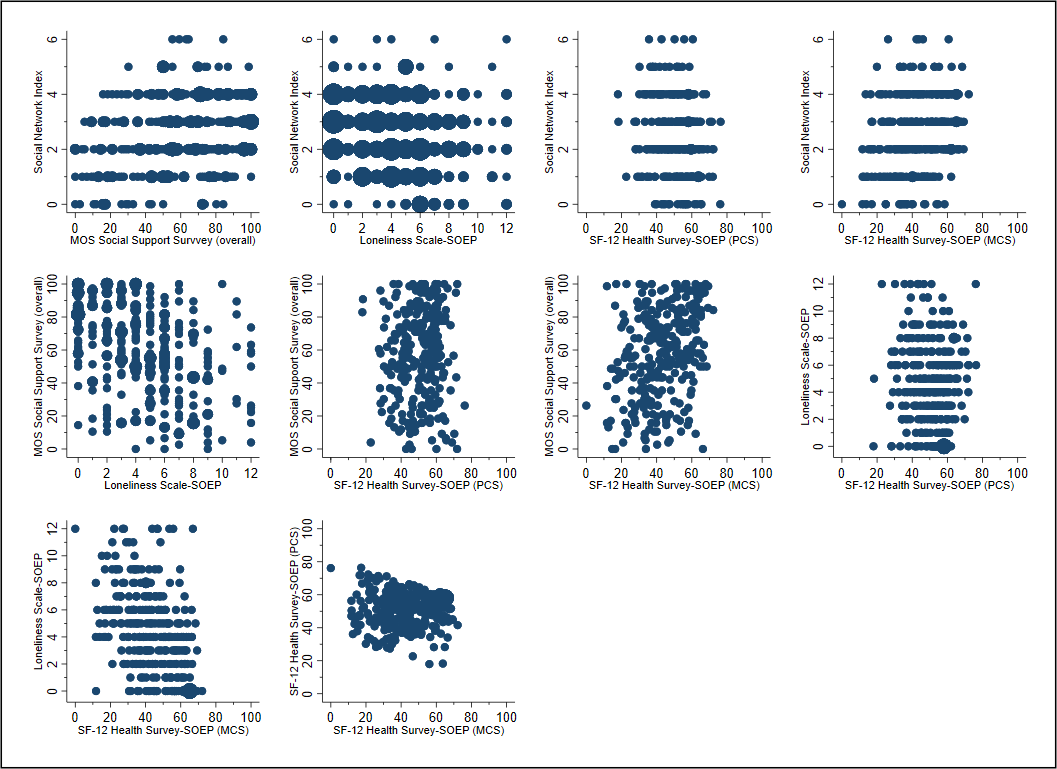

Supplement: Supplementary file 2 — Additional file 2: Figure B. Two-way scatter plots among variables of interest. [file 12889_2021_12303_MOESM2_ESM.tif]
