# Supplementary material for: Loneliness as a mediator of social relationships and health-related quality of life among refugees living in North Rhine-Westphalia, Germany
Source: BMC Public Health. 2021 Dec 8;21:2233. doi: 10.1186/s12889-021-12303-5 (PMC8656054; doi:10.1186/s12889-021-12303-5)
Supplement: Supplementary file 3 — Additional file 3: TableA1. Direct, indirect, and total effects, adjusted for age, sex, and education (n = 268). [file 12889_2021_12303_MOESM3_ESM.docx]

**Table A1:** Direct, indirect and total effects, adjusted for age, sex, and education (N=268)

| **Model pathways** |  | | **ß** | **95% BCa CI** | |  | ***p*-value** |
| --- | --- | --- | --- | --- | --- | --- | --- |
|  |  | |  | **Lower** | **Upper** | |  |
|  |  |  | |  |  | |  |
| **Model 1.1** |  |  | |  |  | |  |
| **Direct effect** |  |  | |  |  | |  |
| Social integration → PCS | c’ | -0.165 | | -0.920 | 0.590 | | 0.668 |
| Social integration → loneliness | a | -0.288 | | -0.561 | -0.015 | | 0.039 |
| Loneliness → PCS | b | -0.621 | | -0.958 | -0.283 | | <0.001 |
| **Indirect effect** |  |  | |  |  | |  |
| Social integration → loneliness → PCS | ab | 0.179 | | -0.015 | 0.372 | | 0.071 |
| **Total effect** |  |  | |  |  | |  |
| Social integration → PCS | c | 0.014 | | -0.786 | 0.813 | | 0.973 |
|  |  |  | |  |  | |  |
| **Model 1.2** |  |  | |  |  | |  |
| **Direct effect** |  |  | |  |  | |  |
| Social integration → MCS | c’ | 2.126 | | 0.944 | 3.307 | | <0.001 |
| Social integration → loneliness | a | -0.288 | | -0.561 | -0.015 | | 0.039 |
| Loneliness → MCS | b | -1.721 | | -2.236 | -1.207 | | <0.001 |
| **Indirect effect** |  |  | |  |  | |  |
| Social integration → loneliness →MCS | ab | 0.495^a^ | | 0.018 | 0.972 | | 0.042 |
| **Total effect** |  |  | |  |  | |  |
| Social integration → MCS | c | 2.621 | | 1.426 | 3.816 | | <0.001 |
|  |  |  | |  |  | |  |
| **Model 2.1** |  |  | |  |  | |  |
| **Direct effect** |  |  | |  |  | |  |
| Social support → PCS | c’ | 0.038 | | -0.008 | 0.083 | | 0.104 |
| Social support → loneliness | a | -0.045 | | -0.058 | -0.033 | | <0.001 |
| Loneliness → PCS | b | -0.490 | | -0.858 | -0.121 | | 0.009 |
| **Indirect effect** |  |  | |  |  | |  |
| Social support → loneliness → PCS | ab | 0.022^a^ | | 0.004 | 0.040 | | 0.017 |
| **Total effect** |  |  | |  |  | |  |
| Social support → PCS | c | 0.060 | | 0.017 | 0.102 | | 0.006 |
|  |  |  | |  |  | |  |
| **Model 2.2** |  |  | |  |  | |  |
| **Direct effect** |  |  | |  |  | |  |
| Social support → MCS | c’ | 0.107 | | 0.039 | 0.174 | | 0.002 |
| Social support → loneliness | a | -0.045 | | -0.058 | -0.032 | | <0.001 |
| Loneliness → MCS | b | -1.493 | | -2.055 | -0.930 | | <0.001 |
| **Indirect effect** |  |  | |  |  | |  |
| Social support → loneliness → MCS | ab | 0.067^a^ | | 0.037 | 0.097 | | <0.001 |
| **Total effect** |  |  | |  |  | |  |
| Social support → MCS | c | 0.174 | | 0.111 | 0.237 | | <0.001 |
|  |  |  | |  |  | |  |
| ß: regression coefficient; 95% BCa CI: bias-corrected and accelerated bootstrapped 95% confidence intervals based on 10,000 samples; ^a^ 95% BCa CI does not contain zero (indirect effect only) | | | | | | | |
